# Extracellular Ribonuclease from *Bacillus licheniformis* (Balifase), a New Member of the N1/T1 RNase Superfamily

**DOI:** 10.1155/2016/4239375

**Published:** 2016-08-30

**Authors:** Yulia Sokurenko, Alsu Nadyrova, Vera Ulyanova, Olga Ilinskaya

**Affiliations:** Institute of Fundamental Medicine and Biology, Kazan Federal University, Kremlevskaya Str. 18, Kazan 420008, Russia

## Abstract

The N1/T1 RNase superfamily comprises enzymes with well-established antitumor effects, such as ribotoxins secreted by fungi, primarily by* Aspergillus* and* Penicillium* species, and bacterial RNase secreted by* B. pumilus *(binase) and* B. amyloliquefaciens* (barnase). RNase is regarded as an alternative to classical chemotherapeutic agents due to its selective cytotoxicity towards tumor cells. New RNase with a high degree of structural similarity with binase (73%) and barnase (74%) was isolated and purified from* Bacillus licheniformis* (balifase, calculated molecular weight 12421.9 Da, pI 8.91). The protein sample with enzymatic activity of 1.5 × 10^6^ units/A_280 _was obtained. The physicochemical properties of balifase are similar to those of barnase. However, in terms of its gene organization and promoter activity, balifase is closer to binase. The unique feature of balifase gene organization consists in the fact that genes of RNase and its inhibitor are located in one operon. Similarly to biosynthesis of binase, balifase synthesis is induced under phosphate starvation; however, in contrast to binase, balifase does not form dimers under natural conditions. We propose that the highest stability of balifase among analyzed RNase types allows the protein to retain its structure without oligomerization.

## 1. Introduction

Ribonuclease (RNase) is involved in many cellular processes including control of gene expression, angiogenesis, apoptosis, and cell defense from pathogens [1–5]. At present, RNase types possessing antitumor and antiviral activities are at the peak of experimental investigation due to their selective toxicity towards cancer or virus-infected cells [3, 6–11]. Among them are secreted RNase types of bacterial origin which belong to N1/T1 (ЕС 3.1.27.3) superfamily. Such RNase types are small (~12.5 kDa) extracellular basic proteins. Upon RNA hydrolysis these enzymes cleave the 3′,5′-phosphodiester bond between guanosine 3′-phosphate and the 5′-OH group of the adjacent nucleotide, while generating 2′,3′-cyclic guanosine phosphate in the first stage of a catalytic reaction. This stage is reversible and faster than the second one, wherein the cyclic intermediate is converted into the corresponding 3′-phosphate derivative [12]. This type of RNase was isolated from the cultural fluid of* B. amyloliquefaciens* (termed barnase, P00648)*, B. pumilus* (binase, P00649),* B. altitudinis* (balnase, A0A0J1IDI7),* B. circulans* (P35078), and* B. coagulans* (P37203). The catalytic activity, molecular structures, and some biological properties of such RNase were explored and characterized [13–17]. The most studied representatives of bacillary RNase are binase and barnase produced by* B. pumilus* and* B. amyloliquefaciens*, respectively. Consisting of ~110 amino acid residues, these enzymes are highly similar in their structure. They also show similar physicochemical and catalytic properties, namely, stability over a wide pH range (3–10) with an optimum at pH 8.5 and nonrequirement in metal ions for ribonucleolytic activity [2].

*Bacillus* spp. are known to produce another type of extracellular RNase with high molecular weight (~30 kDa) and low level of catalytic activity, while lacking specificity towards guanyl residues. These enzymes are exemplified by* B. subtilis* Bsn and* B. pumilus* binase II [18, 19].

*B. licheniformis*, the endospore-forming, nonpathogenic Gram-positive bacteria, is used extensively for industrial production of exoenzymes (proteases, *α*-amylases) and peptide antibiotics [20]. Despite its widespread industrial application, the extracellular RNase from* B. licheniformis* has not yet been characterized. The RNase encoding* BLI_RS18290* gene (previously known as* BLi03719*) was found among the most dominant protein spots in the extracellular proteome of the* B. licheniformis* grown under phosphate deficiency [21].

Here, we have isolated, purified, and characterized the* B. licheniformis* secreted RNase (balifase). Furthermore, we have compared molecular properties of balifase with those of binase and barnase. We have shown that the level of balifase catalytic activity, as well as its physicochemical characteristics and structural features, is similar to those of the main representatives of bacillary N1/T1 RNase. The unique features of balifase include the formation of an operon together with a gene of its intracellular inhibitor, as well as high stability and inability to form natural dimers.

## 2. Materials and Methods

### 2.1. Strain and Growth Conditions

Wild-type strain of* B. licheniformis* ATCC 14580 was obtained from* Bacillus* Genetic Stock Center (BGSC, USA).* B. licheniformis* was grown on standard LB medium or on LP medium (low phosphate peptone: 2%; glucose: 1%; Na_2_HPO_4_: 0.04%; CaCl_2_: 0.01%; MgSO_4_ × 7H_2_O: 0.03%; MnSO_4_: 0.01%; NaCl: 0.3%). Bacteria were cultivated at 37°С using a laboratory shaker with oscillation intensity of 200 rpm (INFORS HT, Switzerland). Culture growth was determined spectrophotometrically at *λ* = 590 nm and expressed as optical density units (OD_590_).

### 2.2. RNase Activity

Determination of RNase activity was performed by the measurement of acid-soluble hydrolysis products of high molecular weight yeast RNA as described earlier [22]. The reaction mixture consisting of enzyme solution, RNA, and 0.25 M Tris-HCl buffer, pH 8.5, was incubated for 15 minutes at 37°С. One unit was defined as the amount of enzyme that increases the extinction of acid-soluble products of RNA hydrolysis at 260 nm per min. Specific activity was calculated as the ratio of the total enzyme activity to the amount of the protein.

### 2.3. Enzyme Preparation

After 24–26 hours of cultivation on LP medium,* B. licheniformis* cells were acidified with acetic acid to pH 5.0, centrifuged at 9000 ×g for 20 minutes at 4°C. The supernatant was diluted twice with sterile distilled water and was applied onto the DEAE-cellulose (Servacel, Germany) column (≈30 mL), equilibrated with 0.01 M Na-acetate buffer, pH 5.0. Then the solution was applied onto the phosphocellulose P-11 (Whatman, England) column (≈50 mL), equilibrated with the same buffer. After that the column was washed with 0.01 M Na-acetate buffer, pH 5.0, until optical density of eluate at 280 nm decreased below 0.05. Then the column was equilibrated with 0.01 M Na-phosphate buffer, pH 7.0. The elution was carried out with 0.2 M Na-phosphate buffer, pH 7.0. Fractions corresponding to RNase activity peak were combined and desalted using centrifugal filter units Ultracel-3K (Merck Millipore, USA). The additional purification was carried out using Biologic DuoFlow FPLC system (BioRad, USA) on the UNOS_6_ (BioRad, USA) column, equilibrated with 20 mM Na-acetate buffer, pH 5.0. Proteins were eluted using a linear gradient of 0-1 M NaCl.

### 2.4. SDS-PAGE and Immunoblotting

Proteins were separated by SDS-PAGE [23] and transferred to a nitrocellulose membrane by Mini Trans-Blot cell (BioRad, USA). For the detection of proteins anti-binase antibodies were used [24]. Visualization of protein bands corresponding to RNase was performed using anti-rabbit IgG-POD secondary antibodies (Sigma-Aldrich, USA) and the LumiLight detection system (Roche Diagnostics, Switzerland).

### 2.5. Zymography

To estimate in-gel RNase activity of proteins we performed zymography analysis as described in [25]. Proteins were separated in 15% polyacrylamide gel with 0.1% SDS (SDS-PAGE) [23]. The resolving gel contained RNA from Torula yeast (Sigma-Aldrich, USA) as a substrate at final concentration of 7 mg/mL. Then the gel was washed with buffer I (10 mM Tris-HCl, 20% isopropanol, pH 7.5) for 10 min to remove SDS and then proteins were refolded by consequent incubation for 10 min in 10 mM Tris-HCl, pH 7.5, and in 100 mM Tris-HCl, pH 7.5. The gel was stained for 10 min with 0.2% toluidine blue (Sigma-Aldrich, USA).

### 2.6. Bioinformatic Analysis

The RNase sequences were extracted from the databases of the National Center for Biotechnology Information NCBI (http://www.ncbi.nlm.nih.gov/). Gene neighborhoods were compared at “Microbes Online” server of the Virtual Institute for Microbial Stress and Survival (http://www.microbesonline.org/). Orthologs of intracellular RNase inhibitor (barstar) were identified with the help of “EDGAR” server (https://edgar.computational.bio.uni-giessen.de/). For multiple alignment of amino acid sequences the program “MUSCLE” (http://www.ebi.ac.uk/Tools/muscle/) was applied. Alignment was carried out on the basis of standard criteria. The software package “MEGA 6.0” was used for construction of phylogenetic trees [26]. Leader peptide of the extracellular RNase of* B. licheniformis* ATCC 14580 was determined using PRED-TAT tool (http://www.compgen.org/tools/PRED-TAT/submit/). Virtual Footprint tool was used for analysis of the transcription factor binding sites [27]. Comparison of physicochemical properties of proteins was performed using ProtParam tool [28]. The three-dimensional structure of balifase was modeled with the help of I-TASSER server without specifying the template [29]. A FATCAT web server was used for flexible structure comparison and structure similarity search (http://fatcat.burnham.org/).

## 3. Results and Discussion

### 3.1. Balifase Is Similar to Barnase by Molecular Properties

**Figure 1:**
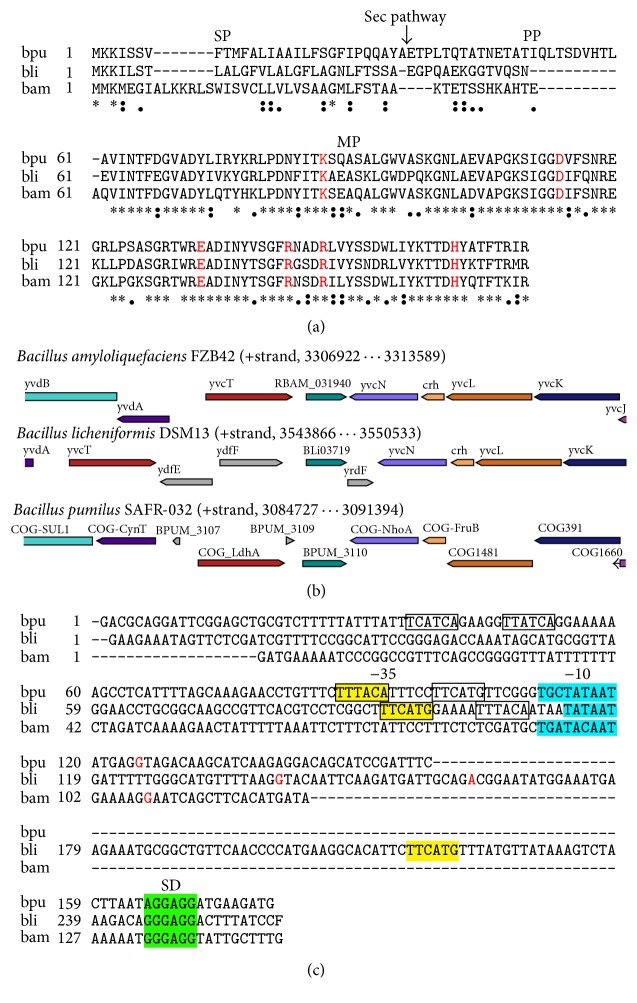
Comparison of* B. licheniformis* RNase with the representatives of N1/T1 RNase family. (a) Sequence alignments of signal peptide (SP), propeptide (PP), and mature peptide (MP) of* B. licheniformis* (bli),* B. amyloliquefaciens* (bam), and* B. pumilus* (bpu) RNase. Identical amino acid residues are marked (*∗*). Amino acid residues that incorporate to the active site of enzyme are red colored. (b) Gene neighborhood of balifase gene in comparison to barnase and binase genes. The data were adopted from the MicrobesOnline Database (http://www.microbesonline.org/). (c) Promoter regions of guanyl-preferring RNase genes from* B. pumilus* (bpu),* B. licheniformis* (bli), and* B. amyloliquefaciens* (bam). Putative PhoP-binding sites are boxed; (+1) regions are red colored. A colon “:” indicates conservation between groups of strongly similar properties. A period “.” indicates conservation between groups of weakly similar properties.

**Figure 2:**
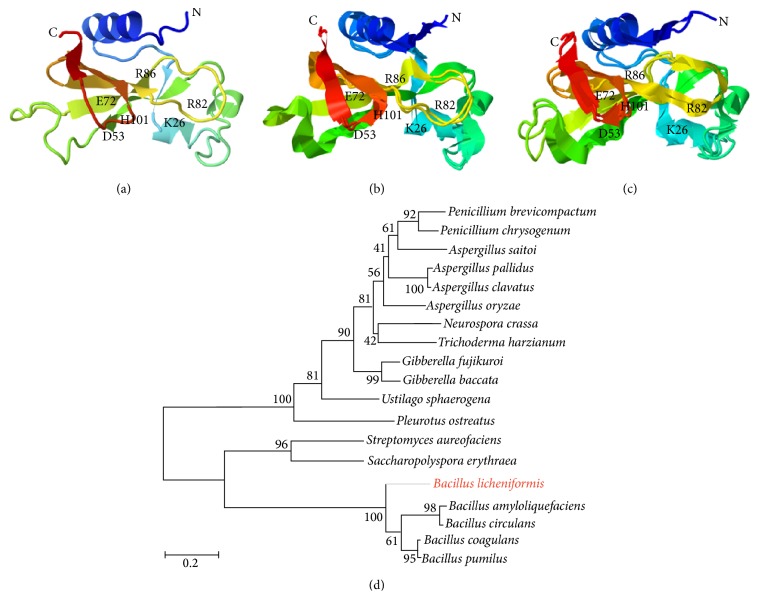
(a) Top model of balifase three-dimensional structure predicted by I-TASSER server without specifying the template. (b) Superimposed three-dimensional structures of binase (PDB ID 1buj) and balifase. (c) Superimposed three-dimensional structures of barnase (PDB ID 1bnr) and balifase. The alignment was performed using the FATCAT server with flexible mode. (d) The phylogenetic tree constructed on the basis of amino acid sequences of RNase from N1/T1 family. The scale bar indicates the average number of amino acid substitutions per site.

To isolate the* B. licheniformis* extracellular RNase which was found upon studies of bacterial cell response to starvation [21], we first compared its gene and amino acid sequence with those of well-studied barnase and binase. The RNase of* B*.* licheniformis* is encoded by the* BLI_RS18290* gene; a mature protein consists of 109 amino acids. The analysis of the primary structure of* B. licheniformis* RNase showed that the main differences of the* B. licheniformis* RNase from binase and barnase are primarily concentrated in the region of the signal and propeptides. The signal peptide of balifase and binase are similar, whereas the propeptide resembles barnase by length. Responsible for transport, maturation, and activation of enzymes, signal peptide may have an effect on spatial organization of proteins. The mature protein is more conserved and differs by 30 and 28 amino acid residues from barnase and binase, respectively (Figure 1(a)). Thus, the RNase of* B. licheniformis* has the 73% and 74% degree of similarity with barnase and binase, respectively. The overall resemblance of the RNase composing the N1/T1 family is reflected in a phylogenetic tree, which was reconstructed based on the primary sequences of the mature RNase (Figure 2(d)). It is shown that the RNase of* B. licheniformis* is equidistant from both* B. amyloliquefaciens* and* B. pumilus* RNase, without forming a single cluster with the latter within the genus.

The three-dimensional structure of balifase was predicted by using the I-TASSER server (Figure 2(a)). The C-score of 1.72 corresponds to a model with high confidence. According to FATCAT pairwise alignment, balifase is very similar to both binase (PDB ID 1buj) and barnase (PDB ID 1bnr) with some minor differences (Figures 2(b) and 2(c)). The *P* value is <0.05 (with the raw score of 298.54 and 282.20, resp.), which means that the structure pairs are significantly similar. The structure alignment has 109 equivalent positions with an RMSD of 1.30 and 1.53, respectively, without twists.

A set of physicochemical parameters of the* B. licheniformis* RNase, such as molecular weight and pI, was predicted by using the ProtParam tool (Table 1). It is shown that despite some variations in these parameters, balifase, binase, and barnase proteins are found to have similar properties. However, balifase is closer to barnase than binase. It is a more acidic protein compared to barnase. Furthermore, the aliphatic index of balifase and barnase is lower than that of binase, which indicates their lower thermostability. The instability index is significantly higher for balifase than for binase and barnase, which points to the high stability of balifase (Table 1). Generally, a protein with an instability index lower than 40 is predicted as stable [30]. Calculated from the molar extinction coefficient of tyrosine, tryptophan, and cystine [31], the extinction coefficient of balifase allows us to detail the calculation of the protein amount during purification.

Therefore, based on the similarities between barnase and balifase proteins, we have found that the protocol for barnase purification is also suitable for purification of balifase [13].

### 3.2. Gene Context of* B. licheniformis* RNase Differs from That of Barnase and Binase

Gene neighborhood information supports a better understanding of putative functions of a protein encoded by a gene of interest. Typically, the neighborhoods combine several genes that are involved in similar process; however, some of these genes can differ. The exploration of the balifase gene* BLI_RS18290* neighborhood showed that it is organized differently from barnase and binase (Figure 1(b)). The RNase gene forms an operon with an intracellular inhibitor gene, downstream of* ydfE* and* ydfF* genes responsible for metal resistance. Yet, these features are not characteristic of binase (*BPUM_3110*) and barnase (*RBAM_031940*). At the 5′-end, they are preceded by the* yvcT* gene encoding gluconate 2-dehydrogenase, as well as the prospective bicistronic operon formed by* yvdA* and* yvdB* genes, which encodes carbonate dehydrogenase and permease of the* SuIP* family. At the 3′-end, the genes for balifase and its inhibitor are followed by* yvcN* (encoding N-acetyltransferase),* crh* (encoding histidine-containing phosphorus-carrying Hpr-like protein),* yvcL* (encoding a DNA-binding protein WhiA, which controls the process of sporulation in spore-forming bacteria),* yvcK* (encoding the factor of gluconeogenesis), and* yvcJ* (encoding a nucleotide-binding protein which hydrolyzes nucleoside triphosphates). The gene context of balifase reflects its participation in phosphorus and carbon metabolism.

The* B. licheniformis* RNase gene context differs from the gene context of other closely related species of bacilli given the fact that balifase gene forms an operon with the gene of intracellular inhibitor YrdF (Figure 1(b)). Our further analysis using the EDGAR server has revealed that the YrdF inhibitor of balifase represents the ortholog of a well-known barnase inhibitor barstar.

The* ydfF* gene encodes a transcriptional regulator, belonging to the family of ArsR-like repressors that activate the transcription of proteins involved in the efflux of metals and/or detoxification by dissociation from operators [32]. The* YdfE* gene product contains flavin reductase domain of various oxidoreductases and monooxygenases. The protein performs an antioxidant function in the oxidation of lipid membrane caused by heavy metals. The expression of the* ydfE* gene is controlled by the* ydfF* gene.

### 3.3. Regulation of Balifase Gene Expression Is Similar to Binase One

To understand how balifase gene expression is regulated we analyzed its promoter structure. Using computational approach it was impossible to identify (−10) and (−35) regions clearly. Two possible (+1) positions were predicted (Figure 1(c)). Therefore, we compared balifase gene promoter to promoters of binase and barnase. The RNase of* Bacillus* with low molecular weight can be divided into two groups: binase-like and barnase-like RNase [33]. In contrast to barnase, the promoters of binase and balifase genes possess (−10) and (−35) regions.

Besides that, the detection of the transcription factor binding sites was performed (Table 2). It was found that expression of balifase, binase, and barnase could be regulated by AbrB, GerE, and PucR transcription factors. This indicates the involvement of the RNase in scavenge of purines (PucR regulation) as well as in general transition from exponential growth to stationary phase (AbrB regulation). Expression of RNase genes during sporulation could be inhibited by GerE. Binding sites for ComK, which is required for the transcription of late competence genes, were not detected in balifase gene promoter in contrast to binase and barnase ones. Genes for barnase and balifase are potentially controlled by DegU, the regulator of extracellular degradative enzymes biosynthesis in response to nitrogen starvation. Furthermore, we identified the potential binding sites for the PhoP transcription factor, which controls cell response to phosphate deficiency in* Bacillus,* in the* B. licheniformis* RNase gene promoter, and in the binase promoter (Figure 1(c)). Potential binding sites for Hpr, which provides the link between phosphorous and carbon metabolism, and SpoIIID, which regulates gene expression during sporulation, were found in binase and balifase gene promoters as well. Thus, analysis of balifase promoter structure and activity revealed its higher similarity to promoter of binase than of barnase.

### 3.4. Purification of Balifase

**Figure 3:**
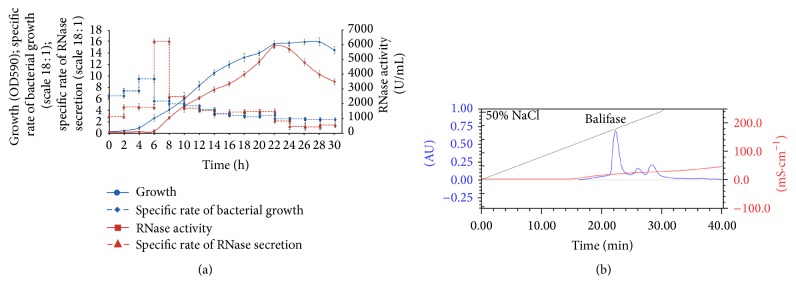
(a) The time-course of* B. licheniformis* ATCC 14580 growth and RNase production on LP medium at 37°C. (b) Elution of balifase fraction in a linear gradient of 0.0–1.0 M NaCl using FPLC Biologic DuoFlow system; AU: absorbance units, mS × cm^−1^: conductivity.

To identify the most suitable media for high-level production of the* B. licheniformis* RNase, we have grown bacteria in LB and low phosphate peptone media (LP) differing by the amount of phosphorus (275 *μ*g/mL and 120 *μ*g/mL, resp.). It was found that the cells produce twice as much amount of RNase when grown on LP medium during 24 h at 37°C. Balifase appears in the culture fluid after 6 h of cultivation and reaches the maximum after 22–24 h corresponding to the early stationary phase (Figure 3(a)).

**Figure 4:**
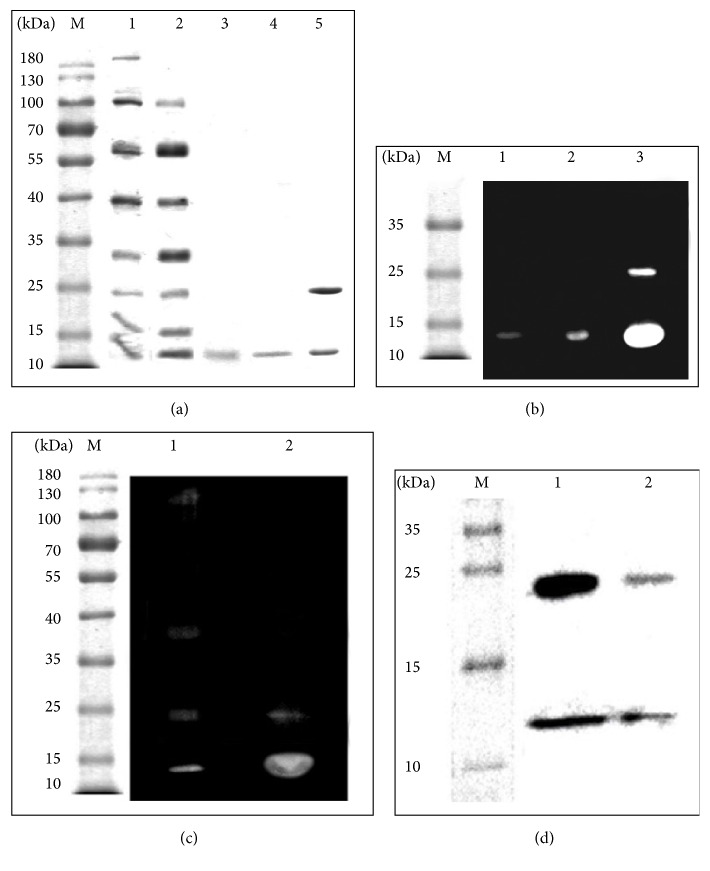
Electrophoretic analysis of purity, enzymatic activity, and antibody specificity of balifase. (a) SDS/PAGE of balifase samples at different stages of purification. 1: before purification in culture fluid, 2: after chromatography on DEAE-cellulose, 3: after chromatography on phosphocellulose P-11, 4: after chromatography on UNOS_6_ column, 5: binase. (b) Zymography analysis of balifase sample. 1: after chromatography on phosphocellulose P-11; 2: after chromatography on UNOS_6_ column; 3: binase. (c) Zymography analysis of balifase sample after concentrating, freezing, and storage. 1: balifase; 2: binase. (d) Western Blot analysis of balifase after chromatography on UNOS_6_ column. 1: binase; 2: balifase.

The purification protocol for barnase [13] was applied for balifase. However, this procedure failed to generate large amounts of pure balifase and, therefore, was modified. For protein purification, samples were collected at 22–24 h and all steps of purification shown in Table 3 were performed. After elution from phosphocellulose, a protein sample with the yield of 75% by activity was obtained. The final purification step was made using the FPLC BioLogic Duo Flow system. Protein was eluted by 0.35 M NaCl (Figure 3(b)). The elution profile was characterized by two additional peaks which had no RNase activity. It was detected via SDS-PAGE that the first fraction contained the protein with the molecular weight of 12 kDa (Figure 4(a)) and RNase activity in the gel (Figure 4(b)). The purity of the sample was shown by mass spectrometry analysis, which also proved the presence of the* B. licheniformis* RNase only (AAU25168.1). Finally, the protein sample with enzymatic activity of 1.5 × 10^6^ units/A_280_ was obtained.

It was observed that the oligomeric forms of balifase with catalytic activity in the gel (Figure 4(c)) appear after concentrating, freezing, and storage. The immunoblot analysis showed that anti-binase antibodies interact with low order oligomers of balifase too (Figure 4(d)). Therefore, we can conclude that balifase can exist in oligomeric forms in solution but unlike binase, not under natural conditions of biosynthesis [25].

## 4. Conclusions

The new low molecular weight RNase isolated from* B. licheniformis* (named balifase) has been characterized according to its physicochemical properties, gene context, and promoter organization. Balifase combines physicochemical properties of barnase (pI, grand average of hydropathicity, and aliphatic index) with the regulatory mode of biosynthesis typical for binase. Both balifase gene and gene for its inhibitor are located in one operon which makes the gene organization of balifase unique. Balifase synthesis is induced under phosphate starvation similarly to biosynthesis of binase; however in contrast to binase balifase does not form dimers under natural conditions. We propose that the highest stability of balifase among analyzed RNase types allows the protein to retain its structure without oligomerization.

## Figures and Tables

**Table 1:** Physicochemical properties of balifase as compared with binase and barnase.

RNase/characteristics	Balifase	Binase	Barnase
Number of amino acids	109	109	110
Molecular weight	12421.9	12211.6	12382.7
Theoretical pI	8.91	9.52	8.88
Extinction coefficient	19940	26930	26930
Abs 0.1% (=1 g/L)	1.605	2.205	2.175
Grand average of hydropathicity	−0.666	−0.416	−0.643
Instability index	8.92	27.25	24.27
Aliphatic index	72.48	78.81	71.00

**Table 2:** Putative binding sites for transcription factors in barnase, binase, and balifase promoters.

Transcription factors	Barnase	Binase	Balifase
*AbrB* (controls the expression of genes involved in starvation-induced processes)	TAAAAAAT (125–132)	GAAAAAAG (54–61)	CAAAAATC (119–126 noncoding strand) CATAAACA (219–226 noncoding strand)
*ComK* (is required for the transcription of the late competence genes)	AAAGAACTATTTT (50–62)	AAAGCCTCATTTT (58–70)	Not detected
*DegU* (regulates the degradative enzyme expression, genetic competence, biofilm formation, and capsule biosynthesis)	GAAAAATCCCGGCCGTTTCAG (4–24)	Not detected	ATAGTTCTCGATCGTTTTCCG (7–27) ACAATAATATAATGATTTTTG (106–126)
*GerE* (regulates gene transcription in the terminally differentiated mother-cell compartment during late stages of sporulation)	AAATGGGAGGTA (129–140)	AAATAAATAAAA (25–36 noncoding strand)	AAATAGTTCTCG (5–16) ATATAATGATTT (112–123)
*Hpr* (provides the link between phosphorous and carbon metabolism)	Not detected	GGTGCTATAATATGAGGTA (109–127)	ATGTTTATGTTATAAAGTC (218–236)
*PucR* (regulation of purine utilization)	ATACAATGAAA (95–105)	ATTCGGAGCTG (9–19)	TTTCATGGAAA (91–101) GTCCTCGGCTT (82–92)
*ResD* (regulation of aerobic and anaerobic respiration)	AACTATTTTTAAA (54–66)	TCATTTTAGCAAA (64–76) CTCATTTTAGCAA (63–75)	AAATTTACAATAA (100–112)
*SpoIIID* (key regulator of transcription during the sporulation process)	Not detected	AGGACAGCAT (141–150) TAGACAAGCA (126–135)	GGCACATTCT (206–215) GGTACAATTC (139–148)

**Table 3:** The isolation and purification of *B. licheniformis* RNase.

Stage of purification	Vol (V), mL	A_280_, units	Specific activity, units/A_280_	Degree of purification	Yield (by activity), %
Culture fluid after 24 h of cultivation	1200	8	763	1	100
After DEAE-cellulose, pH 5.0	1200	7.8	780	1.02	99.7
After elution from phosphocellulose in 200 mM sodium phosphate buffer, pH 7.0	20	0.7	225000	353	75
After chromatography on UNOS_6_ column using FPLC system	4	0.45	1.5 × 10^6^	2353	50
